# HPV Vaccination and Screening with High-Performance Test: Brazilian Evidence

**DOI:** 10.1055/s-0041-1740953

**Published:** 2021-12-21

**Authors:** Julio Cesar Teixeira, Cecilia Maria Roteli-Martins

**Affiliations:** 1 Universidade Estadual de Campinas, Campinas, SP, Brasil; 2 Faculdade de Medicina do ABC, Santo André, SP, Brasil

Brazil has a critical issue on women's health to solve: one woman dies every 90 minutes due to cervical cancer with a mean age of 45 years. 1 Considered eradicable cancer, there are two established strategies to control it: vaccination against HPV and periodic screening for detection of precancerous lesions. The Brazilian public health system offers both, free of charge, although this is not enough.

## Vaccination to Prevent HPV Infection and Cancer

HPV vaccines were globally licensed from 2007–2008 and some Brazilian researchers gave an important contribution to this achievement. Australia, the United Kingdom, Canada, and Sweden, started soon a wide vaccination action reaching large and sustainable coverage in preadolescent and adolescent girls. Their results were presented in recent publications demonstrating the impact over cervical high-grade precursor lesions and cancer incidence, making it possible to project the expected 'elimination' of this cancer. 2 3 4 5

## How was the Winner Strategy? The Answer is School-Based HPV Vaccination

Several other countries, including some considered middle or low-income, are following the same strategy to provide HPV vaccination through schools. Brazil, a continental country with great regional differences, has two main characteristics: the tradition of vaccination with high popular acceptance and a network of elementary-level schools, most of them under municipal administration. Furthermore, the National Immunization Program itself demonstrated in 2014, the same winning strategy and achieved real-life success with 100% coverage for Dose-1. 6

## Why was this Strategy not Continued?

There was probably a lack of central coordination over the various administrative facets involved. Opposing it, any Brazilian municipality can use your regulated autonomy to overcome several of these obstacles. In this issue of RBGO “School-based HPV vaccination: the challenges in a Brazilian initiative,” Teixeira et al. 7 reported the first results of a Brazilian city initiative based on a demonstration study to test this hypothesis: school-based HPV vaccination can increase coverage?

The program started in 2018 and increased three times the Dose-1 coverage in the first year, although had suffered from unexpected obstacles. The authors reported these issues and the strategies applied to overcome them. It should be noted that the problems were not related to safety concerns or acceptance by parents or by education professionals. Even with great interest from local health managers to achieve high coverage vaccination, the program cannot achieve the initial goals, yet. Taking advantage of the moment to disclose partial results and considering data from the period before the pandemic, some strategies adopted in the program are worth highlighting and can be replicated:

1. School-based vaccination for girls and boys aged 9–10 years: nullifying any discussion about gender and prompt access to a high proportion of all children registered in a Municipal school (level 'Fundamental 1'). The city has 87% of all children in this situation.
2. Yearly dose schedule: vaccination once a year facilitates the organization of the health care system to supply vaccination teams to cover all schools. The Dose-2 of the current HPV vaccines has been indicated in six to 12 months intervals, and the risk of interval infection for this early age can be considered insignificant. In addition, studies had demonstrated that the 1-Dose schedule already achieved significant protection. 8

After some unexpected situations limiting the program, the last strategy to ensure the school-based vaccination activity was the passing of a Municipal Law, which determines the availability of a comprehensive structure for school-based vaccination, independent of other competing requests.

## Moving to a High-Performance Screening Testing

The same research team, coordinate another demonstration study to replace the traditional cytological screening for a DNA-HPV test screening, ongoing in the same Brazilian city. Recently, a pivotal cost-effectiveness analysis was published, demonstrating the economic viability of the DNA-HPV testing implementation, with the potential to save resources from the public health perspective. 9 Subsequently, an analysis of the program's early results, still without pandemic interference, has just been published. 10 The researchers pointed to the great potential to save resources and lives when the DNA-HPV testing was applied in a screening with higher coverage, and a higher proportion of the women with abnormal tests evaluated and followed the guidelines. There were demonstrated significant additional cervical cancer cases detected, most of them were prevalent cases, but with an incredible two-thirds proportion of cancer in microinvasive stage, highly curable with more accessible procedures. The prompt impact of organizing the screening program with a high-performance test resulted in anticipating the diagnoses of cervical cancer in 10 years and at early-stage.

In conclusion, the available Brazilian scientific evidence, including databased on real-life, represents to all researchers in this field a kind of mission accomplished. Now, the baton goes over to the next, people who have decision-making on health actions. Our activity, as a medical association, and together with organized society, is to make this information reach the people who decide and to demand them for effective actions.
